# *TERT* promoter methylation is associated with high expression of TERT and poor prognosis in papillary thyroid cancer

**DOI:** 10.3389/fonc.2024.1325345

**Published:** 2024-01-19

**Authors:** Shiyong Li, Junyu Xue, Ke Jiang, Yulu Chen, Lefan Zhu, Rengyun Liu

**Affiliations:** ^1^ Institute of Precision Medicine, The First Affiliated Hospital, Sun Yat-sen University, Guangzhou, China; ^2^ Department of Endocrinology, The First Affiliated Hospital, Sun Yat-sen University, Guangzhou, China; ^3^ Department of Head and Neck Surgery, Sun Yat-sen University Cancer Center, Guangzhou, China

**Keywords:** thyroid cancer, telomerase reverse transcriptase, methylation, prognosis, gene regulation

## Abstract

The telomerase reverse transcriptase (TERT) is overexpressed and associated with poor prognosis in papillary thyroid cancer (PTC), the most common subtype of thyroid cancer. The overexpression of TERT in PTC was partially attributed to transcriptional activation by two hotspot mutations in the core promoter region of this gene. As one of the major epigenetic mechanisms of gene expression regulation, DNA methylation has been proved to regulate several tumor-related genes in PTC. However, the association of *TERT* promoter DNA methylation with TERT expression and PTC progression is still unclear. By treating PTC cell lines with demethylating agent decitabine, we found that the *TERT* promoter methylation and the genes’ expression were remarkably decreased. Consistently, PTC patients with *TERT* hypermethylation had significantly higher TERT expression than patients with *TERT* hypomethylation. Moreover, *TERT* hypermethylated patients showed significant higher rates of poor clinical outcomes than patients with *TERT* hypomethylation. Results from the cox regression analysis showed that the hazard ratios (HRs) of *TERT* hypermethylation for overall survival, disease-specific survival, disease-free interval (DFI) and progression-free interval (PFI) were 4.81 (95% CI, 1.61-14.41), 8.28 (95% CI, 2.14-32.13), 3.56 (95% CI, 1.24-10.17) and 3.32 (95% CI, 1.64-6.71), respectively. The HRs for DFI and PFI remained significant after adjustment for clinical risk factors. These data suggest that promoter DNA methylation upregulates TERT expression and associates with poor clinical outcomes of PTC, thus holds the potential to be a valuable prognostic marker for PTC risk stratification.

## Introduction

Thyroid cancer is the most frequently observed malignancy of the endocrine system (1). Based on histology, thyroid cancer can be divided into four main subtypes, including papillary thyroid cancer (PTC), follicular thyroid cancer (FTC), anaplastic thyroid cancer (ATC), and medullary thyroid cancer (MTC). Among them, PTC has a more favorable prognosis than other subtypes of thyroid cancer (2). However, as the most common subtype, PTC had a sharp increasing incidence in the past three decades and currently accounts for about 90% of all thyroid cases (3).

Overwhelming studies have demonstrated that a number of genetic alterations, including hotspot mutations and fusions in some key cancer driver genes like *BRAF*, *RAS*, *RET*, *EIF1AX* and *telomerase reverse transcriptase* (*TERT*), orchestrated the initiation and progression of thyroid cancer by upregulating the expression of certain oncogenes and downregulating the expression of tumor suppressor genes (4–6). *BRAF* V600E mutation and *RET* fusions are well studied in PTC and it is now well established that they are main drivers of the aberrant MAPK pathway (7, 8). Two hotspot mutations, occurred collectively in around 10% of PTC, in the core promoter region of *TERT* activates the expression of *TERT* at the transcription level and thus promotes the tumorigenesis and development of thyroid cancer (4, 9–11). Clinically, *TERT* promoter mutations had been proved to be strongly associated with aggressive characteristics and poor prognosis of PTC (12–15).

In addition to genetic alterations, epigenetic alterations also involved in the pathogenesis of PTC. As one of the most widely studied epigenetic mechanism, DNA methylation usually observed in the promoter region of tumor suppressor genes (TSGs) and inhibits their expression in human cancers (16). The expression of several classic TSGs, including *RASSF1A*, *CDKN2A* and *DAPK*, and two thyroid-specific genes, *TSHR* and *NIS*, had been identified to be silenced by methylation (17). Interestingly, increasing studies reported that DNA hypermethylation was frequently observed in the promoter region of *TERT* in human cancers and the promoter methylation was emerged as an epigenetic mechanism of TERT activation (18, 19). Clinically, *TERT* hypermethylation was correlated with tumor progression and unfavorable prognosis in several types of cancer (20–23). In this study, we assessed the association of *TERT* promoter methylation with its expression and the clinical outcomes of PTC by analyzing 571 PTC samples in The Cancer Genome Atlas (TCGA) thyroid cancer database, and explored the effect of promoter DNA methylation on TERT expression by demethylating assays in thyroid cancer cell lines.

## Methods

### Clinical data

The clinical data of patients with PTC used in this study was originated from the TCGA thyroid cancer (THCA) dataset. The following clinical factors and prognostic values were collected: age at diagnosis, gender, extrathyroidal extension, pathologic T/N/M, residual tumor, pathologic stage, overall survival (OS), disease-specific survival (DSS), disease-free interval (DFI) and progression-free interval (PFI).

### *TERT* methylation and expression analysis

The β-value, ranging from 0 to 1, was used to show the methylation level of DNA methylation. The β-value of the CpG probe cg11625005 in the promoter of *TERT* and the Illumina HiSeq data of TERT expression for each thyroid sample were downloaded from TCGA dataset through the UCSC Xena platform. If the β-value of cg11625005 of one sample was higher than the mean β-value + 2*SD of the normal samples, this sample was defined as a case with *TERT* hypermethylation.

### Cell line and reagents

Papillary thyroid cancer cell lines BCPAP and TPC1 were purchased from the National Collection of Authenticated Cell Cultures (Shanghai, China) and cultured in RPMI-1640 medium (#10-040-CVRC, Corning) with 10% fetal bovine serum (#04-001-1A, Biological Industries) at 37 °C. DNA demethylating drug decitabine was purchased from Selleck Chemicals (#S1200) and used for treating the two cell lines at 5 μM for 5 days. DMSO was used as the control reagent.

### Genomic DNA preparation and bisulfite sequencing PCR (BSP)

Genomic DNA was extracted from decitabine or DMSO -treated cells using the EZNA Tissue DNA Kit (#D3396-02, Omega Bio-tek) and subjected to sodium bisulfite modification using EZ DNA Methlyation Lightning Kit (#D5031, Zymo Research) according to the manufacturer’s instructions. The *TERT* promoter region was amplified by PCR using ZymoTaq™ PreMix (#E2004, Zymo Research) with two pairs of primers. The primer pair 5’- GGAGGAGGYGGAGTTGGAAGGTGAAGGGGTAGGA-3’ and 5’- CCTCCACATCATAACCCCTCCCTCRAATTACCCCACA-3’ was used for amplifying the -175 to -466 bp of TERT promoter, the primer pair 5’- TGTGGGGTAAttyGAGGGAGGGGttATGATGTGGAGG-3’ and 5’- CCTaaCTCCATTTCCCACCCTTTCTCrAC-3’ was used for amplifying the -430 to -840 region of TERT promoter. The PCR products were then cloned to T vector and subjected to Sanger sequencing.

### RNA extraction and quantitative real-time PCR

Total RNA was extracted from cultured cells using the TRIzol reagent (#15596018, Invitrogen, CA, USA) and reverse-transcribed to cDNA using the RevertAid First Strand cDNA Synthesis Kit (#K1622; ThermoFisher). Gene expression was detected in triplicate using PowerUp SYBR Green Master Mix (#A25742; Applied Biosystem). β-Actin was used as the internal control for normalization. The primers used for TERT cDNA amplification were 5’- GCCTTCAAGAGCCACGTC-3’ (forward) and 5’- CCACGAACTGTCGCATGT-3’ (reverse), the primers for β-Actin were 5’- AGCCTCGCCTTTGCCGA-3’ and 5’- GCGCGGCGATATCATCATC-3’. Relative mRNA expression of TERT was calculated according to the 2^−ΔΔCT^ method.

### Cell viability assay

Two thousand and five hundred BCPAP cells or one thousand TPC1 cells were seeded in triplicate in each well of a 96-well cell culture plate and incubated with DMSO or decitabine at concentrations ranging from 0.1 to 10 uM for 5 days. The cell culture medium and drugs were refreshed daily. At the end of the drug-treated course, the cell viability was determined by the Cell Counting Kit-8 (#K1018, ApexBio) according to the manufacturer’s instructions. The absorbance was measured at 450 nm using a spectrophotometric microplate reader.

### Statistical analysis

All the *in vitro* experiments were performed three times with each done in triplicate. Comparisons of categorical variables were performed using the chi-square test. For continuous variables, the significance of differences between two groups was assessed by the Student’s *t*-test. Kaplan-Meier curves with log-rank tests and Cox regression analysis were used to compare survival data by methylation status. All the statistical analyses were performed by Stata software (version 12.0) and GraphPad Prism (version 8.0). All P values were 2-sided and P<0.05 was considered as statistically significant.

## Results

### Increased TERT expression in PTC patients with *TERT* hypermethylation

**Figure 1:**
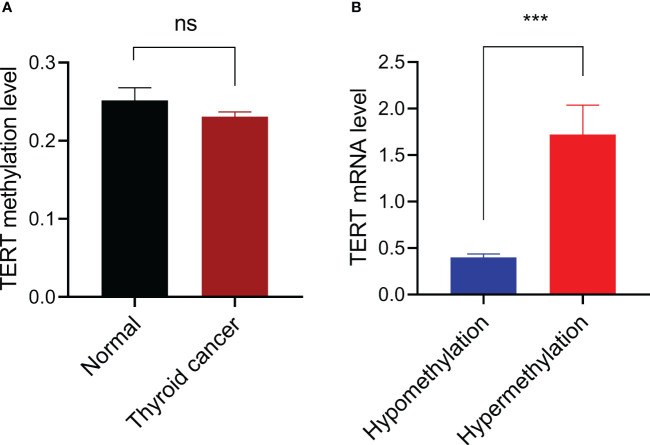
Promoter DNA methylation and TERT expression. **(A)** Comparison of *TERT* promoter methylation levels between 56 thyroid normal tissues and 571 cancer tissues. **(B)** Comparison of TERT mRNA expressions in thyroid cancer samples with *TERT* hypermethylation (n=33) or hypomethylation (n=530). Data were shown as mean ± standard error. ns, not significant; ***P<0.001.

We first compared the *TERT* methylation level in 571 thyroid cancer samples with that in 56 normal thyroid samples and found that there was no significant difference between the two groups (P=0.312, **Figure 1A**). According to our criteria, the cut-off β-value for *TERT* hypermethylation was set at 0.494, and 33 PTC samples were defined as *TERT* hypermethylated cases. Then we analyzed the potential effect of *TERT* methylation on TERT expression by comparing the TERT mRNA levels in *TERT* hypomethylated and hypermethylated thyroid cancer samples. As shown in **Figure 1B**, the patients with *TERT* hypermethylation had a significant high level of TERT expression (1.72 ± 0.32 vs 0.40 ± 0.04, P<0.001).

### Association of *TERT* methylation with clinical characteristics and outcomes of PTC

We next analyzed whether *TERT* methylation was associated with any of the following clinical characteristics of PTC: patient age, gender, extrathyroidal extension, pathologic TNM, residual tumor, and stage. The results showed that there was no significant correlation between *TERT* methylation and any of the clinical characteristics (**Table 1**). However, and importantly, patients with *TERT* hypermethylation had higher rates of poor clinical outcomes than patients with *TERT* hypomethylation (**Table 2**). Specifically, the overall mortality was 4 of 33 (12.1%) in *TERT* hypermethylated patients versus 16 of 538 (3.0%) in *TERT* hypomethylated patients (P=0.023); disease specific mortality was 3 of 32 (9.4%) in *TERT* hypermethylated patients versus 7 of 533 (1.3%) in *TERT* hypomethylated patients (P=0.015); tumor recurrence was 4 of 19 (21.1%) in TERT hypermethylated patients versus 27 of 381 (7.1%) in *TERT* hypomethylated patients (P=0.050); disease progression was observed in 9 of 33 (27.3%) *TERT* hypermethylated patients versus 56 of 538 (10.4%) TERT hypomethylated patients (P=0.003).

**Table 1:** The association of *TERT* methylation with clinical characteristics of PTC.

Characteristic	Overall	Hypermethylation		P
		Yes	No	
No.	571	33	538	
Age	46 (34-58)	51 (33-66)	46 (34-58)	0.082
Gender, male	154/571 (27.0)	12/33 (36.4)	142/538 (26.4)	0.210
Extrathyroidal extension	175/549 (31.9)	11/31 (35.5)	164/518 (31.7)	0.657
Pathologic T				
T1	155 (27.2)	8 (25.0)	147 (27.4)	
T2	191 (33.6)	9 (28.1)	182 (33.9)	0.848
T3	197 (34.6)	12 (37.5)	185 (34.4)	0.708
T4	26 (4.6)	3 (9.4)	23 (4.3)	0.197
Pathologic N	257/515 (49.9)	14/32 (43.8)	243/483 (50.3)	0.472
Pathologic M	11/332 (3.3)	1/20 (5.0)	10/312 (3.2)	0.501
Residual tumor	61/494 (12.3)	3/26 (11.5)	58/468 (12.4)	1.000
Pathologic stage				
I	324 (56.9)	16 (48.5)	308 (57.5)	
II	59 (10.4)	3 (9.1)	56 (10.4)	1.000
III	126 (22.1)	8 (24.2)	118 (22.0)	0.550
IV	60 (10.5)	6 (18.2)	54 (10.1)	0.121

**Table 2:** The association of *TERT* methylation with clinical outcomes of PTC.

Characteristic	Overall, n/N (%)	Hypermethylation, n/N (%)		P
		Yes	No	
All PTC				
Overall mortality	20/571 (3.5)	4/33 (12.1)	16/538 (3.0)	0.023
Disease specific mortality	10/565 (1.8)	3/32 (9.4)	7/533 (1.3)	0.015
Tumor recurrence	31/400 (7.8)	4/19 (21.1)	27/381 (7.1)	0.050
Disease progression	65/571 (11.4)	9/33 (27.3)	56/538 (10.4)	0.003
Stage I/II				
Overall mortality	6/383 (1.6)	0/19 (0)	6/364 (1.6)	1.000
Disease specific mortality	2/382 (0.5)	0/19 (0)	2/363 (0.6)	1.000
Tumor recurrence	20/314 (6.4)	2/14 (14.3)	18/300 (6.0)	0.221
Disease progression	29/383 (7.6)	2/19 (10.5)	27/364 (7.4)	0.646
Stage III/IV				
Overall mortality	14/186 (7.5)	4/14 (28.6)	10/172 (5.8)	0.013
Disease specific mortality	8/181 (4.4)	3/13 (23.1)	5/168 (3.0)	0.013
Tumor recurrence	11/86 (12.8)	2/5 (40.0)	9/81 (11.1)	0.121
Disease progression	36/186 (19.4)	7/14 (50.0)	29/172 (16.9)	0.007

**Figure 2:**
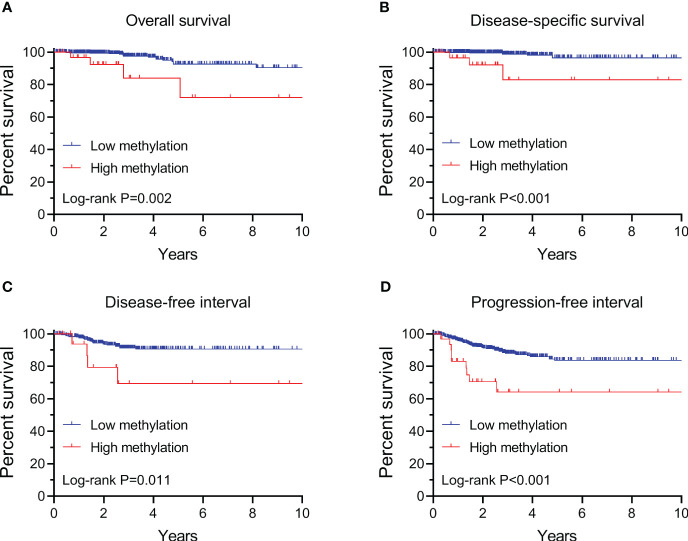
Kaplan-Meier analysis of the association of *TERT* methylation with clinical outcomes of all PTC. Results of the analysis for overall survival **(A)**, disease-specific survival **(B)**, disease-free interval **(C)** and progression-free interval **(D)**. The P values labeled in each panel were calculated by log-rank tests.

**Figure 3:**
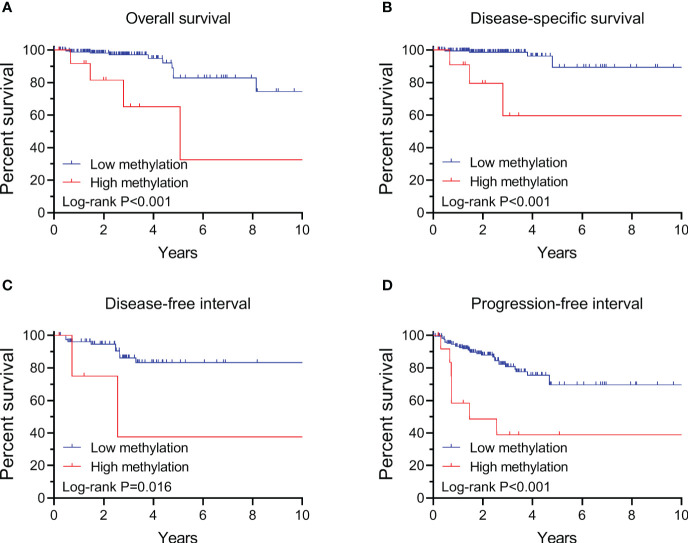
Kaplan-Meier analysis of the association of *TERT* methylation with clinical outcomes of advanced PTC (stage III/IV). Results of the analysis for overall survival **(A)**, disease-specific survival **(B)**, disease-free interval **(C)** and progression-free interval **(D)**. The P values labeled in each panel were calculated by log-rank tests.

By performing Kaplan-Meier and cox regression analyses, we further assessed the impact of *TERT* methylation on the prognosis of PTC. The overall survival (OS) curves had a modest decline in patients with *TERT* hypomethylation, and declined more sharper in patients with *TERT* hypermethylation (**Figure 2A**). As shown in **Figures 2B–D**, similar patterns were obtained when analyzing disease-specific survival (DSS), disease-free interval (DFI) and progression-free interval (PFI). Results from the cox regression analysis showed that the HRs of TERT hypermethylation for OS and DSS were 4.81 (95% CI, 1.61-14.41) and 8.28 (95% CI, 2.14-32.13), respectively. These significances were lost after adjustment for multiple risk factors of PTC. The HRs of *TERT* hypermethylation for DFI and PFI were 3.56 (95% CI, 1.24-10.17) and 3.32 (95% CI, 1.64-6.71), respectively, and they remained significant after multiple variables adjustment (**Table 3**). Then we stratified all the PTC samples into two groups according to the tumor stage, and found that *TERT* hypermethylation was associated poor clinical outcomes in advanced PTC patients (stage III/IV, **Tables 2**, **4** and **Figure 3**).

**Table 3:** Hazard ratios of *TERT* methylation for clinical outcomes of all PTC.

	1000-person years	Crude HR (95% CI)	Adjusted HR (95% CI)*
OS			
Low methylation	8.55 (5.24-13.95)	1.00	1.00
High methylation	40.65 (15.26-108.31)	4.81 (1.60-14.41)	2.42 (0.65-9.06)
DSS			
Low methylation	3.74 (1.78-7.85)	1.00	1.00
High methylation	30.49 (9.83-94.53)	8.28 (2.14-32.13)	2.28 (0.34-15.36)
DFI			
Low methylation	20.11 (13.79-29.32)	1.00	1.00
High methylation	62.04 (23.28-165.30)	3.56 (1.24-10.17)	3.72 (1.28-10.81)
PFI			
Low methylation	32.00 (24.62-41.58)	1.00	1.00
High methylation	102.75 (53.46-197.48)	3.32 (1.64-6.71)	2.66 (1.24-5.67)

*Adjusted for age at diagnosis, sex, extrathyroidal extension, pathologic T and N. OS, overall survival; DSS, disease specific survival; DFI, disease free interval; PFI, progression free interval.

**Table 4:** Hazard ratios of *TERT* methylation for clinical outcomes of advanced PTC.

	1000-person years	Crude HR (95% CI)	Adjusted HR (95% CI)*
OS			
Low methylation	21.67 (12.30-38.15)	1.00	1.00
High methylation	50.92 (26.49-97.85)	5.86 (1.83-18.77)	2.94 (0.75-11.49)
DSS			
Low methylation	10.83 (4.87-24.11)	1.00	1.00
High methylation	45.26 (22.63-90.50)	9.00 (2.14-37.77)	4.51 (0.63-32.29)
DFI			
Low methylation	35.00 (18.21-67.27)	1.00	1.00
High methylation	120.68 (30.18-482.54)	5.38 (1.15-25.14)	4.99 (0.94-26.55)
PFI			
Low methylation	64.50 (45.62-91.21)	1.00	1.00
High methylation	125.33 (79.94-196.48)	3.95 (1.73-9.04)	2.83 (1.10-7.33)

*Adjusted for age at diagnosis, sex, extrathyroidal extension, pathologic T and N. OS, overall survival; DSS, disease specific survival; DFI, disease free interval; PFI, progression free interval.

### Demethylating agent treatment decreased TERT expression and cell viability in *TERT* hypermethylated PTC cells

**Figure 4:**
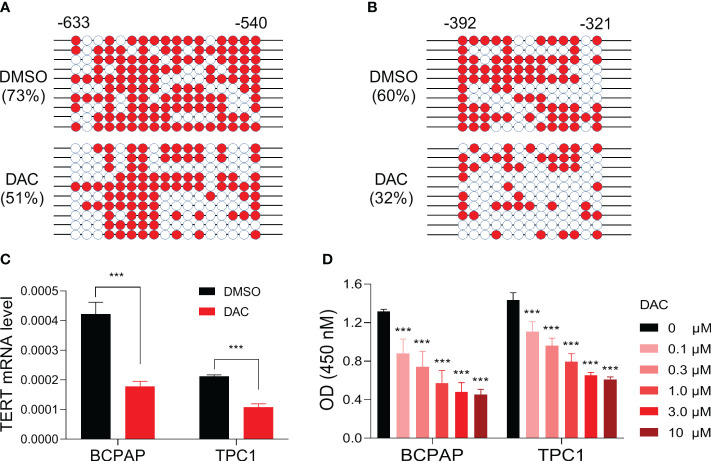
Impact of demethylating treatment on TERT expression and cell viability in PTC cell lines. DNA methylation levels of *TERT* promoter region regions -633 to -540 **(A)** and -392 to -321 **(B)** in BCPAP cells treated with DMSO or 5 uM of decitabine for 5 days. The methylated clones were shown with red color, and the unmethylated clones were shown with white color. **(C)** TERT mRNA levels in PTC cell lines treated with or without decitabine. The relative mRNA expression of TERT was determined by qPCR. **(D)** The absorbances of cells treated with DMSO or decitabine at various doses as indicated. DAC, decitabine. ***P<0.001.

To determine whether *TERT* promoter methylation regulates the gene’s expression in PTC, we selected BCPAP, a PTC cell line with *TERT* hypermethylation (24), as a model and treated it with the classic demethylation agent decitabine and then checked the changes of *TERT* methylation and mRNA expression. As show in **Figures 4A, B**, after treatment with the demethylating agent, the methylation levels of *TERT* promoter regions -633 to -540 and -392 to -321 were decreased from 73% to 51% and from 60% to 32%, respectively. Importantly, the mRNA expression of *TERT* was significantly decreased after decitabine treatment (**Figure 4C**). Next, we tested whether DNA demethylation affects the growth of thyroid cancer cells and found that decitabine decreased the cell viability of BCPAP cells in a dose-dependent manner (**Figure 4D**). Similarly, significant TERT mRNA downregulation and cell growth inhibition were observed when another PTC cell line TPC1 was treated by decitabine (**Figures 4C, D**). These data suggest that promoter DNA methylation positively regulates TERT expression in PTC cells and demethylation treatment suppresses the growth of PTC cells.

## Discussion

*TERT* is regarded as a key oncogene in the tumorigenesis and development of human cancer. It is the catalytic subunit of telomerase which is essential for maintaining the length of telomere in cancer cell immortalization (25). And increasingly recent studies identified that *TERT* is also involved in cancer progression in a telomere-independent manner. For example, knockdown of *TERT* induced quick inhibition of cell proliferation, migration and invasion, while overexpression of TERT variants that lack telomerase activities promotes cell proliferation (26–28). In addition, TERT regulates NF-κB -dependent gene expression by binding to the promoter region of these genes, suppresses TGF-β -dependent growth suppression, and function as a central regulator of various hallmark features of cancer, including resistance to apoptosis, angiogenesis, metabolism reprogramming, genome instability (29, 30).

TERT is overexpressed in thyroid cancer and associated with poor prognosis of PTC (31). Previous studies had shown that *TERT* promoter mutation is one of the major contributors to the gene’s overexpression in thyroid cancer (32–34). Molecular studies further revealed that the two mutations in *TERT* promoter generated consensus binding sites for ETS transcription factors and thereby promoted *TERT* activation (4, 35–37). A large number of genetic association studies have demonstrated that *TERT* promoter mutation is associated with multiple characteristics of aggressive tumor behaviors and patients with *TERT* promoter mutation had a higher risk of recurrence and mortality (12–15, 38).

In this study we found that there is no significant difference of *TERT* promoter methylation level between normal thyroid tissues and PTC tissues, suggesting that promoter DNA methylation had little contribution to the overall increase of TERT overexpression in PTC. It should be noticed that the average level of *TERT* methylation is low in thyroid tissues, therefore we next defined hypermethylation in the tumor samples and obtained 33 of 531 (5.8%) *TERT* hypermethylated samples. Compared with patients carrying hypomethylated *TERT*, the *TERT* hypermethylated patients had significantly higher TERT expression. These data suggest that hypermethylation in the *TERT* promoter is likely to positively regulate TERT expression.

To confirm this, we assessed the effect of DNA methylation on TERT expression in a cell line model. After treating the PTC cells with demethylating agent decitabine, we found that the methylation level of *TERT* promoter was remarkably decreased and the expression of TERT mRNA was correspondingly decreased significantly. To the best of our knowledge, this is the first experimental evidence to show that *TERT* promoter DNA methylation positively regulates TERT expression in PTC. Our finding is consistent with previous report that the DNA methylation in the *TERT* hypermethylated oncological region upregulates the promoter’s activity (18). There are at least three possible mechanisms underlying the methylation mediated TERT activation. First, the transcriptional activator GSC showed the most remarkable binding in PTC cells than other cell lines, suggesting GSC acts as a putative regulator for methylated *TERT* in PTC (24). Second, allele-specific regulation of TERT was proposed to explain its activation in human cancer, and an interplay between *TERT* promoter mutation, methylation, and histone modification were involved in this process (24, 39–42). Third, a 1.6 kb antisense long non-coding RNA, named hTERT antisense promoter-associated (hTAPAS), in the *TERT* promoter region was proved to regulate TERT expression through DNA methylation in urothelial cancer (43). As the epigenetic regulation on gene expression is usually occurred in a tissue type dependent manner, whether the methylation of hTAPAS involves TERT regulation in PTC needs to be further pursued.

The associations between *TERT* methylation and clinical tumor behaviors and outcomes of thyroid cancer had been investigated by few preliminary studies with limited number of thyroid patients (44, 45). In this study we comprehensively analyzed the correlation between *TERT* methylation and clinical outcomes of PTC by enrolling 571 samples from the TCGA thyroid cancer database, and found that patients with *TERT* hypermethylation had unfavorable OS, DSS, DFI and PFI. Importantly, the association between *TERT* hypermethylation and DFI and PFI is still significant after adjusting multiple clinical parameters. These data strongly indicated that DNA hypermethylation in *TERT* promoter is associated with poor prognosis of PTC, and it could be serving as a prognostic marker for risk stratification of PTC. Similarly, the prognostic role of *TERT* promoter methylation had been reported in several other types of cancer, including childhood brain tumors (20), pancreatic cancer (21), bladder cancer (22), and breast cancer (23).

In conclusion, our data showed that DNA hypermethylation in the promoter region of *TERT* upregulated TERT expression in PTC, and *TERT* promoter hypermethylated patients with PTC had a poor clinical outcome. Our findings in this study indicate a prognostic value of *TERT* methylation in PTC and needs to be validated in further studies.

## Data availability statement

The publicly available TCGA data were analyzed and the TCGA thyroid cancer datasets used in this study were available at the Xena TCGA data hub (https://xenabrowser.net/datapages/?cohort=TCGA%20Thyroid%20Cancer%20(THCA)).

## Ethics statement

Ethical approval was not required for the studies on humans in accordance with the local legislation and institutional requirements because only commercially available established cell lines were used.

## Author contributions

SL: Methodology, Resources, Writing – original draft, Data curation, Formal analysis, Investigation, Writing – review & editing. JX: Investigation, Data curation, Formal analysis, Methodology, Writing – review & editing. KJ: Writing – review & editing, Data curation, Formal analysis, Investigation, Methodology. YC: Writing – review & editing, Data curation, Investigation, Methodology. LZ: Writing – review & editing, Data curation, Investigation, Methodology. RL: Writing – review & editing, Conceptualization, Data curation, Formal analysis, Funding acquisition, Investigation, Methodology, Project administration, Resources, Supervision, Writing – original draft.

## References

[1] Miranda-Filho A Lortet-Tieulent J Bray F Cao B Franceschi S Vaccarella S. Thyroid cancer incidence trends by histology in 25 countries: a population-based study. Lancet Diabetes Endocrinol (2021) 9(4):225–34. doi: 10.1016/S2213-8587(21)00027-9 33662333

[2] Cabanillas ME McFadden DG Durante C. Thyroid cancer. Lancet (2016) 388(10061):2783–95. doi: 10.1016/S0140-6736(16)30172-6 27240885

[3] Lim H Devesa SS Sosa JA Check D Kitahara CM. Trends in thyroid cancer incidence and mortality in the United States, 1974-2013. JAMA (2017) 317(13):1338–48. doi: 10.1001/jama.2017.2719 PMC8216772 28362912

[4] Liu R Zhang T Zhu G Xing M. Regulation of mutant TERT by BRAF V600E/MAP kinase pathway through FOS/GABP in human cancer. Nat Commun (2018) 9(1):579. doi: 10.1038/s41467-018-03033-1 29422527 PMC5805723

[5] Krishnamoorthy GP Davidson NR Leach SD Zhao Z Lowe SW Lee G. EIF1AX and RAS Mutations Cooperate to Drive Thyroid Tumorigenesis through ATF4 and c-MYC. Cancer Discovery (2019) 9(2):264–81. doi: 10.1158/2159-8290.CD-18-0606 PMC6373451 30305285

[6] Fagin JA Wells SA Jr. Biologic and clinical perspectives on thyroid cancer. N Engl J Med (2016) 375(11):1054–67. doi: 10.1056/NEJMra1501993 PMC5512163 27626519

[7] Xing M. BRAF mutation in papillary thyroid cancer: pathogenic role, molecular bases, and clinical implications. Endocr Rev (2007) 28(7):742–62. doi: 10.1210/er.2007-0007 17940185

[8] Salvatore D Santoro M Schlumberger M. The importance of the RET gene in thyroid cancer and therapeutic implications. Nat Rev Endocrinol (2021) 17(5):296–306. doi: 10.1038/s41574-021-00470-9 33603219

[9] Landa I Ganly I Chan TA Mitsutake N Matsuse M Ibrahimpasic T. Frequent somatic TERT promoter mutations in thyroid cancer: higher prevalence in advanced forms of the disease. J Clin Endocrinol Metab (2013) 98(9):E1562–6. doi: 10.1210/jc.2013-2383 PMC3763971 23833040

[10] Liu R Xing M. TERT promoter mutations in thyroid cancer. Endocr Relat Cancer (2016) 23(3):R143–55. doi: 10.1530/ERC-15-0533 PMC4750651 26733501

[11] Liu X Bishop J Shan Y Pai S Liu D Murugan AK. Highly prevalent TERT promoter mutations in aggressive thyroid cancers. Endocr Relat Cancer (2013) 20(4):603–10. doi: 10.1530/ERC-13-0210 PMC3782569 23766237

[12] Melo M da Rocha AG Vinagre J Batista R Peixoto J Tavares C. TERT promoter mutations are a major indicator of poor outcome in differentiated thyroid carcinomas. J Clin Endocrinol Metab (2014) 99(5):E754–65. doi: 10.1210/jc.2013-3734 PMC4191548 24476079

[13] Xing M Liu R Liu X Murugan AK Zhu G Zeiger MA. BRAF V600E and TERT promoter mutations cooperatively identify the most aggressive papillary thyroid cancer with highest recurrence. J Clin Oncol (2014) 32(25):2718–26. doi: 10.1200/JCO.2014.55.5094 PMC4145183 25024077

[14] Kim TH Kim YE Ahn S Kim JY Ki CS Oh YL. TERT promoter mutations and long-term survival in patients with thyroid cancer. Endocr Relat Cancer (2016) 23(10):813–23. doi: 10.1530/ERC-16-0219 27528624

[15] Liu R Bishop J Zhu G Zhang T Ladenson PW Xing M. Mortality risk stratification by combining BRAF V600E and TERT promoter mutations in papillary thyroid cancer: genetic duet of BRAF and TERT promoter mutations in thyroid cancer mortality. JAMA Oncol (2017) 3(2):202–8. doi: 10.1001/jamaoncol.2016.3288 27581851

[16] Baylin SB. DNA methylation and gene silencing in cancer. Nat Clin Pract Oncol (2005) 2 Suppl 1:S4–11. doi: 10.1038/ncponc0354 16341240

[17] Zafon C Gil J Perez-Gonzalez B Jorda M. DNA methylation in thyroid cancer. Endocr Relat Cancer (2019) 26(7):R415–R39. doi: 10.1530/ERC-19-0093 31035251

[18] Lee DD Leao R Komosa M Gallo M Zhang CH Lipman T. DNA hypermethylation within TERT promoter upregulates TERT expression in cancer. J Clin Invest (2019) 129(1):223–9. doi: 10.1172/JCI121303 PMC6307937 30358567

[19] Lee DD Komosa M Nunes NM Tabori U. DNA methylation of the TERT promoter and its impact on human cancer. Curr Opin Genet Dev (2020) 60:17–24. doi: 10.1016/j.gde.2020.02.003 32114294

[20] Castelo-Branco P Choufani S Mack S Gallagher D Zhang C Lipman T. Methylation of the TERT promoter and risk stratification of childhood brain tumours: an integrative genomic and molecular study. Lancet Oncol (2013) 14(6):534–42. doi: 10.1016/S1470-2045(13)70110-4 23598174

[21] Faleiro I Apolonio JD Price AJ De Mello RA Roberto VP Tabori U. The TERT hypermethylated oncologic region predicts recurrence and survival in pancreatic cancer. Future Oncol (2017) 13(23):2045–51. doi: 10.2217/fon-2017-0167 29019414

[22] Leao R Lee D Figueiredo A Hermanns T Wild P Komosa M. Combined genetic and epigenetic alterations of the TERT promoter affect clinical and biological behavior of bladder cancer. Int J Cancer (2019) 144(7):1676–84. doi: 10.1002/ijc.31935 PMC6519346 30350309

[23] Apolonio JD Dias JS Fernandes MT Komosa M Lipman T Zhang CH. THOR is a targetable epigenetic biomarker with clinical implications in breast cancer. Clin Epigenet (2022) 14(1):178. doi: 10.1186/s13148-022-01396-3 PMC9759897 36529814

[24] Avin BA Wang Y Gilpatrick T Workman RE Lee I Timp W. Characterization of human telomerase reverse transcriptase promoter methylation and transcription factor binding in differentiated thyroid cancer cell lines. Genes Chromosomes Cancer (2019) 58(8):530–40. doi: 10.1002/gcc.22735 PMC6621557 30664813

[25] Blasco MA. Telomeres and human disease: ageing, cancer and beyond. Nat Rev Genet (2005) 6(8):611–22. doi: 10.1038/nrg1656 16136653

[26] Smith LL Coller HA Roberts JM. Telomerase modulates expression of growth-controlling genes and enhances cell proliferation. Nat Cell Biol (2003) 5(5):474–9. doi: 10.1038/ncb985 12717449

[27] Choi J Southworth LK Sarin KY Venteicher AS Ma W Chang W. TERT promotes epithelial proliferation through transcriptional control of a Myc- and Wnt-related developmental program. PLoS Genet (2008) 4(1):e10. doi: 10.1371/journal.pgen.0040010 18208333 PMC2211538

[28] Hrdlickova R Nehyba J Bose HR Jr. Alternatively spliced telomerase reverse transcriptase variants lacking telomerase activity stimulate cell proliferation. Mol Cell Biol (2012) 32(21):4283–96. doi: 10.1128/MCB.00550-12 PMC3486134 22907755

[29] Ghosh A Saginc G Leow SC Khattar E Shin EM Yan TD. Telomerase directly regulates NF-kappaB-dependent transcription. Nat Cell Biol (2012) 14(12):1270–81. doi: 10.1038/ncb2621 23159929

[30] Low KC Tergaonkar V. Telomerase: central regulator of all of the hallmarks of cancer. Trends Biochem Sci (2013) 38(9):426–34. doi: 10.1016/j.tibs.2013.07.001 23932019

[31] Tanaka A Matsuse M Saenko V Nakao T Yamanouchi K Sakimura C. TERT mRNA expression as a novel prognostic marker in papillary thyroid carcinomas. Thyroid (2019) 29(8):1105–14. doi: 10.1089/thy.2018.0695 31286848

[32] Vinagre J Almeida A Populo H Batista R Lyra J Pinto V. Frequency of TERT promoter mutations in human cancers. Nat Commun (2013) 4:2185. doi: 10.1038/ncomms3185 23887589

[33] Yuan X Larsson C Xu D. Mechanisms underlying the activation of TERT transcription and telomerase activity in human cancer: old actors and new players. Oncogene (2019) 38(34):6172–83. doi: 10.1038/s41388-019-0872-9 PMC6756069 31285550

[34] Panebianco F Nikitski AV Nikiforova MN Nikiforov YE. Spectrum of TERT promoter mutations and mechanisms of activation in thyroid cancer. Cancer Med (2019) 8(13):5831–9. doi: 10.1002/cam4.2467 PMC6792496 31408918

[35] Song YS Yoo SK Kim HH Jung G Oh AR Cha JY. Interaction of BRAF-induced ETS factors with mutant TERT promoter in papillary thyroid cancer. Endocr Relat Cancer (2019) 26(6):629–41. doi: 10.1530/ERC-17-0562 30999281

[36] Bullock M Lim G Zhu Y Aberg H Kurdyukov S Clifton-Bligh R. ETS factor ETV5 activates the mutant telomerase reverse transcriptase promoter in thyroid cancer. Thyroid (2019) 29(11):1623–33. doi: 10.1089/thy.2018.0314 31452441

[37] Thornton CEM Hao J Tamarapu PP Landa I. Multiple ETS factors participate in the transcriptional control of TERT mutant promoter in thyroid cancers. Cancers (Basel) (2022) 14(2):357. doi: 10.3390/cancers14020357 35053525 PMC8774187

[38] Matsuse M Mitsutake N. TERT promoter mutations in thyroid cancer. Endocr J (2023) 70(11):1035-49. doi: 10.1507/endocrj.EJ23-0136 37532521

[39] McKelvey BA Umbricht CB Zeiger MA. Telomerase reverse transcriptase (TERT) regulation in thyroid cancer: A review. Front Endocrinol (Lausanne) (2020) 11:485. doi: 10.3389/fendo.2020.00485 32849278 PMC7412884

[40] Stern JL Paucek RD Huang FW Ghandi M Nwumeh R Costello JC. Allele-specific DNA methylation and its interplay with repressive histone marks at promoter-mutant TERT genes. Cell Rep (2017) 21(13):3700–7. doi: 10.1016/j.celrep.2017.12.001 PMC5747321 29281820

[41] Rowland TJ Bonham AJ Cech TR. Allele-specific proximal promoter hypomethylation of the telomerase reverse transcriptase gene (TERT) associates with TERT expression in multiple cancers. Mol Oncol (2020) 14(10):2358–74. doi: 10.1002/1878-0261.12786 PMC7530785 33245585

[42] McKelvey BA Zeiger MA Umbricht CB. Exploring the epigenetic regulation of telomerase reverse transcriptase (TERT) in human cancer cell lines. Mol Oncol (2020) 14(10):2355–7. doi: 10.1002/1878-0261.12798 PMC7530778 32920953

[43] Ott P Arauzo-Bravo MJ Hoffmann MJ Poyet C Bendhack ML Santourlidis S. Differential DNA Methylation of THOR and hTAPAS in the Regulation of hTERT and the Diagnosis of Cancer. Cancers (Basel) (2022) 14(18):4384. doi: 10.3390/cancers14184384 36139544 PMC9497117

[44] Li JJ Zheng PCJ Wang YZ. The correlations between DNA methylation and polymorphisms in the promoter region of the human telomerase reverse transcriptase (hTERT) gene with postoperative recurrence in patients with thyroid carcinoma (TC). World J Surg Oncol (2017) 15(1):114. doi: 10.1186/s12957-017-1170-z 28587656 PMC5461729

[45] Montero-Conde C Leandro-Garcia LJ Martinez-Montes AM Martinez P Moya FJ Leton R. Comprehensive molecular analysis of immortalization hallmarks in thyroid cancer reveals new prognostic markers. Clin Transl Med (2022) 12(8):e1001. doi: 10.1002/ctm2.1001 35979662 PMC9386325

